# Supplementary material for: A SNP-based genome-wide association study (GWAS) of seed-yield related traits in Psathyrostachys juncea using wheat as a reference genome
Source: PeerJ. 2025 Jul 22;13:e19617. doi: 10.7717/peerj.19617 (PMC12292311; doi:10.7717/peerj.19617)
Supplement: Supplemental Information 1 [file peerj-13-19617-s001.docx]

**Table S1.** Geographic information of *P. juncea* populations

| **Sample ID** | **Individual plant number** | **Sample number** | **Origin** | **Germplasm preservation institutions** | **Cultivation** |
| --- | --- | --- | --- | --- | --- |
| PI 531828 | 1-14 | 14 | Idaho, U.S. | NPGS, U.S. | Wild |
| PI 595135 | 15-28 | 14 | Xin jiang,China | NPGS, U.S. | Wild |
| PI 619487 | 29-42 | 14 | Mongolia | NPGS, U.S. | Wild |
| CF 005043 | 43-56 | 14 | China | National Medium term Gene Bank of Forage Germplasm, China | Cultivated |
| PI 502577 | 57-70 | 14 | Russian Federation | NPGS, U.S. | Cultivated |
| PI 549118 | 71-84 | 14 | Utah, U.S. | NPGS, U.S. | Cultivated |
| PI 502576 | 85-98 | 14 | Russian Federation | NPGS, U.S. | Cultivated |
| PI 565060 | 99-112 | 14 | Russian Federation | NPGS, U.S. | Wild |
| PI 565051 | 113-123 | 11 | Russian Federation | NPGS, U.S. | Wild |
| PI 578854 | 124-138 | 15 | Saskatchewan, Canada | NPGS, U.S. | Cultivated |
| PI 619483 | 139-153 | 15 | Mongolia | NPGS, U.S. | Wild |
| PI 565052 | 154-167 | 14 | Russian Federation | NPGS, U.S. | Wild |
| PI 531827 | 168-182 | 15 | Estonia | NPGS, U.S. | Wild |
| PI 272136 | 183-195 | 13 | Alma-Asa, Kazakhstan | NPGS, U.S. | Cultivated |
| PI 502573 | 196-210 | 15 | Former Soviet Union | NPGS, U.S. | Cultivated |
| PI 598614 | 211-224 | 14 | Kazakhstan | NPGS, U.S. | Wild |
| PI 476299 | 225-237 | 13 | North Dakota, U.S. | NPGS, U.S. | Cultivated |
| PI 619565 | 238-251 | 14 | Mongolia | NPGS, U.S. | Wild |
| PI 531826 | 252-268 | 17 | China | NPGS, U.S. | Wild |
| PI 502572 | 269-284 | 16 | Former Soviet Union | NPGS, U.S. | Cultivated |
| PI 598610 | 285-300 | 16 | Kazakhstan | NPGS, U.S. | Wild |

**Table S2.** General environmental parameters of the test locations

| **Factors** | **Hohhot** | | **Baotou** | |
| --- | --- | --- | --- | --- |
|  | 2021 | 2022 | 2021 | 2022 |
| Mean annual precipitation (mm) | 390.7 | 249.0 | 240.6 | 239.1 |
| Daily mean temp (^o^C) | 7.7 | 7.7 | 8.4 | 8.1 |
| Relative humidity (%) | 49.1 | 47 | 57.8 | 55 |
| Mean effective sunshine hours (h) | 2727.4 | 2907.7 | 2978.9 | 3102.8 |
| Soil pH | 7.60 | | 8.00 | |
| Soil organic carbon (g**·**kg^-1^) | 11.98 | | 15.44 | |
| Total nitrogen (g**·**kg^-1^) | 0.97 | | 1.69 | |
| Soil available phosphorus (mg**·**kg^-1^) | 20.54 | | 6.37 | |
| Soil rapidly available potassium (mg**·**kg^-1^) | 171.8 | | 205.6 | |

**Table S3.** Five population structure groups differing in eight traits in the population across two environments

|  | **RTL** | **RTN** | **SL** | **SW** | **SNN** | **TKW** | **SWS** | **SY** |
| --- | --- | --- | --- | --- | --- | --- | --- | --- |
| Group 1 | 115.93 ^a^ | 88 ^a^ | 11.87 ^a^ | 0.67 ^a^ | 33 ^a^ | 0.204 ^a^ | 0.287 ^a^ | 24.915 ^a^ |
| Group 2 | 112.77 ^a^ | 68 ^b^ | 11.70 ^b^ | 0.63 ^cd^ | 31 ^a^ | 0.200 ^b^ | 0.275 ^a^ | 19.003 ^b^ |
| Group 3 | 114.66 ^a^ | 65 ^bc^ | 10.84 ^d^ | 0.64 ^b^ | 30 ^b^ | 0.197 ^b^ | 0.261 ^b^ | 18.873 ^c^ |
| Group 4 | 108.24^b^ | 57 ^d^ | 10.86 ^d^ | 0.63 ^d^ | 28 ^b^ | 0.196 ^b^ | 0.246 ^d^ | 14.670 ^e^ |
| Group 5 | 114.34 ^a^ | 61 ^c^ | 11.16 ^c^ | 0.64 ^bc^ | 30 ^a^ | 0.206 ^a^ | 0.254 ^c^ | 16.211 ^d^ |

Note: Means followed by the same letter or not followed by any letter within a column for a given treatment were not significantly different at *p* < 0.05.

Table S4. Significant associations of SNP with eight traits in the population.

| **Trait** | **SNP** | **alleles** | **Chromosome** | **Position** | ***p*-values** | **Count** | **MAF** | ***R*2（%）** | **Environment** | **Reference** | **Physical positions** |
| --- | --- | --- | --- | --- | --- | --- | --- | --- | --- | --- | --- |
| RTL | Chr2A_72607643 | G/A | 2A* | 72607643 | 8.68E-10 | G:498_A:48 | 0.09 | 10.13~14.32 | E1/E2/E3/E4/B | Kuang 2020 | 73.3Mb |
|  | Chr2D_386256188 | T/A | 2D | 386256188 | 9.03E-07 | T:59_A:541 | 0.1 | 6.15~9.21 | E1/E3 |  |  |
|  | Chr4A_216071736 | A/G | 4A | 216071736 | 6.09E-07 | A:31_G:471 | 0.06 | 5.32~8.45 | E1 |  |  |
|  | Chr4B_364667161 | G/A | 4B | 364667161 | 1.26E-06 | G:407_A:171 | 0.3 | 6.92~7.21 | E1 |  |  |
|  | Chr5D_370786992 | C/T | 5D | 370786992 | 1.28E-06 | C:524_T:34 | 0.06 | 5.24~9.13 | E1 |  |  |
|  | Chr6B_221817913 | A/G | 6B | 221817913 | 4.76E-08 | A:32_G:552 | 0.05 | 8.32~9.93 | E1/E2 |  |  |
|  | Chr6D_132666605 | G/A | 6D | 132666605 | 6.52E-07 | G:501_A:33 | 0.06 | 7.34~9.32 | E1/E3 |  |  |
| RTN | Chr1B_602637178 | C/T | 1B | 602637178 | 9.23E-07 | C:459_T:63 | 0.12 | 9.21~9.82 | E2/E4/B |  |  |
|  | Chr2A_88665738 | C/G | 2A | 88665738 | 4.71E-07 | C:533_G:67 | 0.11 | 5.54~8.31 | E1/E2 |  |  |
|  | Chr2B_150707314 | A/G | 2B | 150707314 | 3.18E-07 | A:42_G:530 | 0.07 | 10.11~10.54 | E1/E3 |  |  |
|  | Chr2B_508951798 | T/C | 2B | 508951798 | 3.78E-07 | T:173_C:375 | 0.32 | 8.31~10.32 | E1 |  |  |
|  | Chr2B_710021947 | C/T | 2B | 710021947 | 2.36E-08 | C:139_T:461 | 0.23 | 9.21~10.32 | E1/E2/B |  |  |
|  | Chr2D_133335759 | T/C | 2D | 133335759 | 1.18E-06 | T:28_C:484 | 0.05 | 8.31~9.99 | E2 |  |  |
|  | Chr2D_544686276 | T/A | 2D | 544686276 | 4.88E-07 | T:54_A:484 | 0.1 | 7.43~9.12 | E2 |  |  |
|  | Chr2D_640215049 | T/G | 2D* | 640215049 | 3.73E-09 | T:554_G:34 | 0.06 | 10.41~11.22 | E1/E2/E3/E4/B |  |  |
|  | Chr3B_244391331 | T/C | 3B | 244391331 | 3.79E-07 | T:33_C:495 | 0.06 | 8.31~8.91 | E1/E3 |  |  |
|  | Chr3B_802748211 | T/C | 3B | 802748211 | 7.87E-07 | T:197_C:365 | 0.35 | 9.21~9.91 | E1/E3 |  |  |
|  | Chr3B_815941709 | A/G | 3B | 815941709 | 1.23E-06 | A:110_G:478 | 0.19 | 8.21~10.22 | E3/E4 |  |  |
|  | Chr3D_387780034 | G/A | 3D | 387780034 | 1.12E-06 | G:35_A:555 | 0.06 | 5.30~9.31 | E1 |  |  |
|  | Chr4A_29187500 | C/T | 4A | 29187500 | 2.08E-07 | C:463_T:27 | 0.06 | 4.33~6.98 | E2/E3/E4 |  |  |
|  | Chr4A_722894581 | T/C | 4A* | 722894581 | 1.30E-09 | T:84_C:488 | 0.15 | 9.97~11.22 | E1/E2/E3/E4/B | Cui 2014 | 724.7Mb |
|  | Chr4B_10453308 | T/A | 4B | 10453308 | 9.21E-07 | T:47_A:517 | 0.08 | 5.44~7.21 | E3/E4 |  |  |
|  | Chr5A_520644886 | T/C | 5A | 520644886 | 8.85E-07 | T:50_C:534 | 0.09 | 5.92~8.24 | E3/E4/B |  |  |
|  | Chr5A_595391324 | T/G | 5A | 595391324 | 1.21E-06 | T:465_G:25 | 0.05 | 9.21~9.90 | E3 |  |  |
|  | Chr5B_203504750 | C/T | 5B | 203504750 | 2.80E-08 | C:562_T:32 | 0.05 | 8.91~9.32 | E2/E4/B |  |  |
|  | Chr5D_332563850 | T/C | 5D | 332563850 | 8.22E-08 | T:29_C:473 | 0.06 | 5.21~8.32 | E1/E2 |  |  |
|  | Chr6D_433535351 | T/C | 6D | 433535351 | 5.87E-06 | T:38_C:530 | 0.07 | 6.19~8.35 | E1/E2 |  |  |
|  | Chr6D_433535431 | A/G | 6D | 433535431 | 9.50E-07 | A:530_G:38 | 0.07 | 5.32~7.34 | E1/E2 |  |  |
|  | Chr7A_144661625 | C/T | 7A | 144661625 | 3.96E-07 | C:490_T:46 | 0.09 | 5.84~7.33 | E2/E4/B |  |  |
|  | Chr7A_675095747 | G/T | 7A* | 675095747 | 2.59E-09 | G:439_T:41 | 0.09 | 10.33~12.44 | E1/E2/E3/E4/B | Cao 2020 | 675Mb |
|  | Chr7D_110633650 | G/A | 7D | 110633650 | 2.94E-08 | G:458_A:28 | 0.06 | 9.22~10.11 | E1/E2/E3 |  |  |
| SL | Chr1A_324564082 | C/T | 1A | 324564082 | 1.95E-07 | C:512_T:30 | 0.06 | 5.82~9.33 | E2 |  |  |
|  | Chr1A_522952279 | C/G | 1A | 522952279 | 2.41E-07 | C:39_G:481 | 0.07 | 8.43~9.22 | E2/E3/E4 |  |  |
|  | Chr1D_123176259 | C/G | 1D | 123176259 | 4.72E-09 | C:480_G:28 | 0.06 | 5.92~8.33 | E1 |  |  |
|  | Chr1D_285425889 | A/T | 1D | 285425889 | 2.35E-08 | A:550_T:40 | 0.07 | 6.22~8.38 | E1 |  |  |
|  | Chr2A_170450385 | T/A | 2A | 170450385 | 1.56E-08 | T:472_A:34 | 0.07 | 5.21~7.29 | E1 |  |  |
|  | Chr2A_766154600 | G/A | 2A | 766154600 | 2.88E-08 | G:489_A:31 | 0.06 | 6.33~8.32 | E1/E3 |  |  |
|  | Chr2B_322449354 | G/A | 2B | 322449354 | 3.72E-09 | G:441_A:39 | 0.08 | 5.32~7.33 | E1/E3/B |  |  |
|  | Chr2D_111603346 | T/C | 2D | 111603346 | 1.82E-09 | T:501_C:27 | 0.05 | 9.31~10.22 | E1/E2/B |  |  |
|  | Chr2D_142447607 | C/T | 2D | 142447607 | 8.07E-09 | C:524_T:38 | 0.07 | 4.52~9.32 | E1 |  |  |
|  | Chr2D_427634147 | G/A | 2D | 427634147 | 6.05E-08 | G:492_A:28 | 0.05 | 8.43~9.46 | E1 |  |  |
|  | Chr2D_637673537 | G/A | 2D | 637673537 | 1.29E-06 | G:363_A:173 | 0.32 | 7.34~9.32 | E1 |  |  |
|  | Chr2D_650894588 | G/A | 2D* | 650894588 | 2.42E-13 | G:471_A:29 | 0.06 | 9.31~12.32 | E1/E2/E3/E4 |  |  |
|  | Chr3A_186838870 | C/T | 3A | 186838870 | 1.14E-07 | C:496_T:40 | 0.07 | 5.43~7.44 | E2/E4/B |  |  |
|  | Chr3A_694940541 | C/T | 3A | 694940541 | 6.93E-09 | C:466_T:26 | 0.05 | 8.87~10.32 | E1/E2 |  |  |
|  | Chr3B_559095345 | C/T | 3B | 559095345 | 6.85E-08 | C:446_T:42 | 0.09 | 5.43~8.34 | E2/E4 |  |  |
|  | Chr3B_731265195 | C/A | 3B | 731265195 | 5.07E-09 | C:471_A:25 | 0.05 | 6.43~9.43 | E2/E4 |  |  |
|  | Chr3D_8915769 | C/T | 3D | 8915769 | 7.03E-08 | C:448_T:36 | 0.07 | 6.45~7.96 | E1/E2 |  |  |
|  | Chr3D_458311131 | G/A | 3D | 458311131 | 3.88E-08 | G:507_A:29 | 0.05 | 5.87~7.69 | E1 |  |  |
|  | Chr3D_568444945 | C/T | 3D | 568444945 | 3.93E-08 | C:486_T:32 | 0.06 | 6.12~8.44 | E1 |  |  |
|  | Chr4A_602917167 | G/A | 4A | 602917167 | 8.00E-08 | G:530_A:44 | 0.08 | 6.21~8.25 | E1/E2/E3 |  |  |
|  | Chr4B_253711646 | A/C | 4B | 253711646 | 1.99E-08 | A:524_C:32 | 0.06 | 7.11~7.89 | E2/B |  |  |
|  | Chr4B_518083232 | G/C | 4B | 518083232 | 7.75E-07 | G:33_C:491 | 0.06 | 4.56~7.85 | E1 |  |  |
|  | Chr4D_177570461 | C/T | 4D | 177570461 | 5.65E-08 | C:510_T:38 | 0.07 | 4.44~8.52 | E2/E3/E4 |  |  |
|  | Chr5A_25613052 | C/T | 5A | 25613052 | 1.59E-08 | C:476_T:26 | 0.05 | 5.36~8.12 | E2/E3 |  |  |
|  | Chr5A_657544633 | C/G | 5A* | 657544633 | 8.31E-10 | C:48_G:496 | 0.09 | 9.11~13.54 | E1/E2/E3/E4/B |  |  |
|  | Chr5B_249093155 | A/T | 5B | 249093155 | 1.26E-09 | A:545_T:29 | 0.05 | 6.23~8.11 | E3/E4 |  |  |
|  | Chr5B_359414644 | C/T | 5B | 359414644 | 2.49E-08 | C:535_T:29 | 0.05 | 5.96~7.45 | E3/E4/B |  |  |
|  | Chr5B_544659547 | G/T | 5B | 544659547 | 6.39E-09 | G:460_T:26 | 0.05 | 8.12~9.44 | E3/E4/B |  |  |
|  | Chr5B_544659583 | G/C | 5B | 544659583 | 2.02E-09 | G:455_C:25 | 0.05 | 5.61~8.11 | E3 |  |  |
|  | Chr5D_339963450 | G/C | 5D | 339963450 | 7.86E-09 | G:539_C:43 | 0.07 | 6.12~7.45 | E3 |  |  |
|  | Chr5D_340983074 | T/C | 5D | 340983074 | 3.23E-09 | T:478_C:28 | 0.06 | 5.13~8.74 | E3 |  |  |
|  | Chr5D_367242097 | A/G | 5D | 367242097 | 3.21E-07 | A:25_G:465 | 0.05 | 5.14~7.89 | E3 |  |  |
|  | Chr5D_405156269 | C/T | 5D* | 405156269 | 9.54E-10 | C:525_T:35 | 0.06 | 10.69~14.51 | E1/E2/E3/E4/B |  |  |
|  | Chr5D_500222088 | T/C | 5D | 500222088 | 6.96E-08 | T:540_C:38 | 0.07 | 6.47~7.85 | E2 |  |  |
|  | Chr5D_537909393 | G/T | 5D | 537909393 | 5.12E-09 | G:466_T:36 | 0.07 | 7.98~9.87 | E2/E3 |  |  |
|  | Chr6A_17541092 | G/A | 6A* | 17541092 | 1.38E-11 | G:490_A:26 | 0.05 | 10.21~14.58 | E1/E2/E3/E4 |  |  |
|  | Chr6A_46515260 | G/A | 6A | 46515260 | 1.22E-07 | G:511_A:69 | 0.12 | 6.57~7.89 | E1/E2 |  |  |
|  | Chr6D_102238677 | C/A | 6D | 102238677 | 2.37E-09 | C:503_A:29 | 0.05 | 8.54~9.81 | E1 |  |  |
|  | Chr7A_65973829 | G/T | 7A | 65973829 | 9.71E-08 | G:492_T:26 | 0.05 | 7.54~8.97 | E1/E2 |  |  |
|  | Chr7A_459001661 | T/C | 7A | 459001661 | 4.18E-08 | T:458_C:28 | 0.06 | 6.54~9.89 | E1/E2/E3 |  |  |
|  | Chr7A_675095747 | G/T | 7A* | 675095747 | 2.98E-11 | G:439_T:41 | 0.09 | 10.87~15.64 | E1/E2/E3/E4 | Gill 2022 | 676Mb |
|  | Chr7B_47240012 | T/A | 7B | 47240012 | 1.89E-08 | T:494_A:36 | 0.07 | 4.68~7.89 | E1/E2 |  |  |
|  | Chr7B_130817143 | C/T | 7B | 130817143 | 1.29E-08 | C:510_T:34 | 0.06 | 6.78~8.97 | E1 |  |  |
|  | Chr7B_152457372 | A/T | 7B | 152457372 | 8.53E-09 | A:524_T:32 | 0.06 | 6.45~8.89 | E1/E2/E3 |  |  |
|  | Chr7B_463032072 | A/T | 7B* | 463032072 | 2.50E-10 | A:491_T:27 | 0.05 | 8.54~11.87 | E1/E2/E3/E4/B | Li 2020 | 458Mb |
|  | Chr7D_6779494 | G/A | 7D | 6779494 | 3.31E-07 | G:34_A:526 | 0.06 | 6.89~9.41 | E3/E4 |  |  |
|  | Chr7D_32598993 | T/G | 7D | 32598993 | 4.04E-09 | T:464_G:28 | 0.06 | 5.98~9.61 | E3/E4 |  |  |
|  | Chr7D_146385788 | A/T | 7D | 146385788 | 3.29E-09 | A:531_T:47 | 0.08 | 6.57~8.47 | E1/E2 |  |  |
| SW | Chr3D_126230806 | T/C | 3D | 126230806 | 5.68E-07 | T:43_C:473 | 0.08 | 7.84~8.91 | E2 |  |  |
|  | Chr4A_722894581 | T/C | 4A* | 722894581 | 1.30E-10 | T:84_C:488 | 0.15 | 10.96~13.45 | E1/E2/E3/E4/B |  |  |
|  | Chr4B_548104537 | G/C | 4B | 548104537 | 2.95E-07 | G:500_C:54 | 0.1 | 5.68~7.74 | E1/E2 |  |  |
|  | Chr5A_657544633 | C/G | 5A* | 657544633 | 8.31E-10 | C:48_G:496 | 0.09 | 10.94~13.47 | E1/E2/E3/E4/B | Shui 2020 | 657.5Mb |
|  | Chr5B_279098432 | G/A | 5B | 279098432 | 9.26E-07 | G:48_A:464 | 0.09 | 4.65~7.68 | E2/E3 |  |  |
|  | Chr7B_500234600 | T/C | 7B | 500234600 | 9.30E-07 | T:29_C:533 | 0.05 | 6.54~9.89 | E2/E3/E4 |  |  |
|  | Chr7B_500234809 | T/A | 7B | 500234809 | 1.11E-06 | T:530_A:32 | 0.06 | 7.54~8.97 | E2/E4 |  |  |
| SNN | Chr1A_522952279 | C/G | 1A | 522952279 | 1.79E-07 | C:39_G:481 | 0.07 | 9.12~10.21 | E2/E4/B |  |  |
|  | Chr1D_355520490 | C/T | 1D | 355520490 | 1.53E-07 | C:547_T:45 | 0.08 | 5.64~7.45 | E2 |  |  |
|  | Chr1D_485326317 | T/A | 1D | 485326317 | 3.88E-07 | T:76_A:514 | 0.13 | 5.67~7.98 | E2 |  |  |
|  | Chr7A_675095747 | G/T | 7A* | 675095747 | 2.49E-09 | G:439_T:41 | 0.09 | 8.19~13.14 | E1/E2/E3/E4 | Voss-Fels 2019 | 674Mb |
|  | Chr7A_283122975 | A/G | 7A | 283122975 | 6.71E-07 | A:36_G:554 | 0.06 | 6.54~7.98 | E1/E2 |  |  |
|  | Chr7D_6779494 | G/A | 7D | 6779494 | 1.33E-07 | G:34_A:526 | 0.06 | 6.12~7.89 | E1/E3/B |  |  |
| TKW | Chr2B_452997948 | C/T | 2B | 452997948 | 1.14E-06 | C:31_T:553 | 0.05 | 6.14~8.74 | E1/E2 |  |  |
|  | Chr2D_387683982 | T/C | 2D | 387683982 | 1.09E-06 | T:63_C:435 | 0.13 | 6.94~8.84 | E3/E4 |  |  |
|  | Chr2D_640215049 | T/G | 2D* | 640215049 | 1.68E-11 | T:554_G:34 | 0.06 | 10.14~14.51 | E1/E2/E3/E4/B | Cao 2020 | 640.5Mb |
|  | Chr4A_599779031 | G/A | 4A | 599779031 | 6.81E-07 | G:567_A:33 | 0.06 | 4.15~6.17 | E1/E2 |  |  |
|  | Chr5B_382776118 | A/G | 5B | 382776118 | 5.34E-07 | A:488_G:26 | 0.05 | 5.14~6.47 | E1/E2 |  |  |
|  | Chr6A_582234607 | T/C | 6A | 582234607 | 3.98E-08 | T:527_C:33 | 0.06 | 10.31~14.21 | E1/E3/B |  |  |
|  | Chr7A_675095747 | G/T | 7A* | 675095747 | 9.11E-11 | G:439_T:41 | 0.09 | 11.31~14.51 | E1/E2/E3/E4/B | Guan 2018 | 675Mb |
|  | Chr7D_264317510 | G/A | 7D | 264317510 | 4.98E-07 | G:27_A:479 | 0.05 | 6.41~8.22 | E2/E3/E4 |  |  |
|  | Chr7D_392213206 | G/A | 7D | 392213206 | 7.72E-07 | G:486_A:114 | 0.19 | 5.74~7.64 | E2/E4 |  |  |
| SWS | Chr2A_72607643 | G/A | 2A* | 72607643 | 5.56E-09 | G:498_A:48 | 0.09 | 9.14~11.95 | E1/E2/E3/E4/B |  |  |
|  | Chr2B_531159718 | A/G | 2B | 531159718 | 7.73E-07 | A:31_G:477 | 0.06 | 6.12~9.84 | E2/E4/B |  |  |
|  | Chr3B_380728057 | C/T | 3B | 380728057 | 4.65E-07 | C:220_T:380 | 0.37 | 6.84~9.14 | E2/E4 |  |  |
|  | Chr4A_174990264 | C/G | 4A | 174990264 | 1.61E-07 | C:428_G:146 | 0.25 | 8.14~9.98 | E2/E4 |  |  |
|  | Chr4D_133474511 | T/C | 4D | 133474511 | 5.83E-07 | T:38_C:558 | 0.06 | 6.51~9.87 | E2 |  |  |
|  | Chr5A_608956411 | T/C | 5A | 608956411 | 5.36E-09 | T:44_C:440 | 0.09 | 8.74~9.88 | E2 |  |  |
|  | Chr5A_608956414 | T/C | 5A | 608956414 | 2.77E-08 | T:440_C:42 | 0.09 | 6.51~9.41 | E2/B |  |  |
|  | Chr5A_608956425 | C/T | 5A | 608956425 | 3.96E-09 | C:428_T:56 | 0.12 | 6.58~8.45 | E2/E4 |  |  |
|  | Chr5A_608956441 | A/G | 5A | 608956441 | 7.16E-08 | A:64_G:420 | 0.13 | 6.11~8.44 | E2/E3/E4 |  |  |
|  | Chr5D_405156269 | C/T | 5D* | 405156269 | 3.26E-10 | C:525_T:35 | 0.06 | 10.36~14.45 | E1/E2/E3/E4 | Liu 2023 | 403.05Mb |
|  | Chr6A_582234607 | T/C | 6A | 582234607 | 7.40E-08 | T:527_C:33 | 0.06 | 10.51~11.56 | E1/E3/B |  |  |
|  | Chr6D_225221107 | G/A | 6D | 225221107 | 1.20E-06 | G:457_A:125 | 0.21 | 6.57~10.54 | E1/E2 | Liu 2023 | 24.04Mb |
|  | Chr7B_500234600 | T/C | 7B | 500234600 | 1.31E-06 | T:29_C:533 | 0.05 | 7.45~8.54 | E1 |  |  |
|  | Chr7D_626351918 | A/G | 7D | 626351918 | 1.26E-06 | A:517_G:31 | 0.06 | 5.46~7.51 | E1 |  |  |
| SY | Chr2B_710021947 | C/T | 2B | 710021947 | 2.71E-08 | C:139_T:461 | 0.23 | 5.67~9.54 | E2/E4 |  |  |
|  | Chr2D_650894588 | G/A | 2D* | 650894588 | 4.72E-11 | G:471_A:29 | 0.06 | 13.11~14.51 | E1/E2/E3/E4 | Li 2018a | 650.7Mb |
|  | Chr4D_98916589 | A/C | 4D | 98916589 | 4.81E-08 | A:37_C:469 | 0.07 | 6.54~9.14 | E1/E2/B |  |  |
|  | Chr5B_203504750 | C/T | 5B | 203504750 | 6.34E-08 | C:562_T:32 | 0.05 | 6.45~7.93 | E2/E4 |  |  |
|  | Chr6A_17541092 | G/A | 6A* | 17541092 | 3.21E-10 | G:490_A:26 | 0.05 | 10.21~12.45 | E1/E2/E3/E4/B | Fei 2022 | 17.6Mb |
|  | Chr7D_110633650 | G/A | 7D | 110633650 | 6.23E-08 | G:458_A:28 | 0.06 | 8.51~9.88 | E1/E2/B |  |  |

Note: Indicates Bonferroni corrected *P* value (*P* valve < 1.0E-06), SNPs are in complete LD. Hohhot and Baotou location in 2021, and Hohhot and Baotou location in 2022 are represented by E1-E4, respectively; B represents BLUP; * Represents significant SNPs detected in four different environments.





**Figure S1.** Bar chart for the seed yield related traits phenotypic values of eight different genetypes of eight SNPs

Note: (a): Chr2A_72607643; (b): Chr2D_640215049; (c): Chr2D_650894588; (d): Chr4A_722894581; (e): Chr5A_657544633; (f): Chr5D_405156269; (g): Chr6A_17541092; (h): Chr7A_675095747


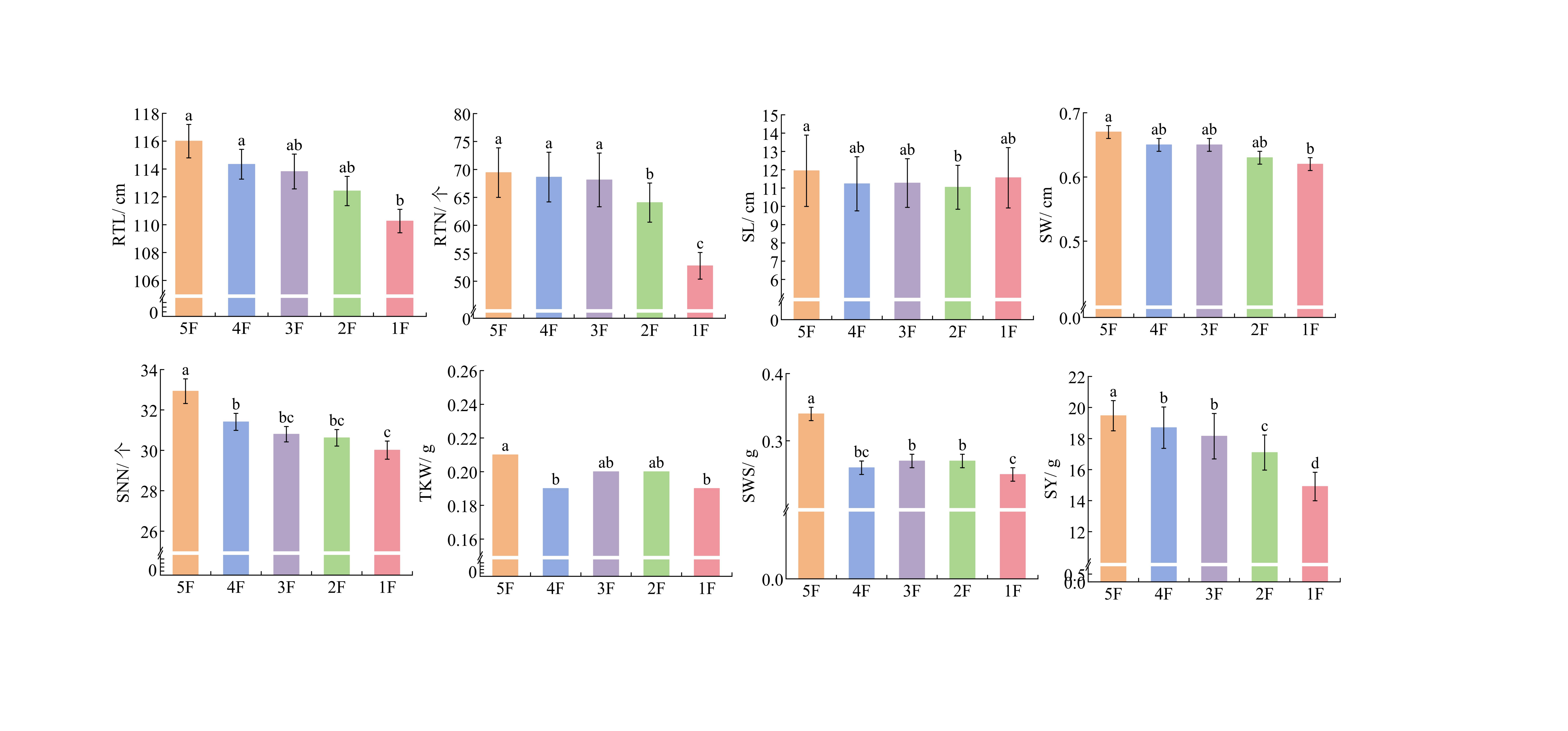


### **Figure S2** Pyramiding effects of the favorable genetype alleles that contribute to seed yield related traits
